# Primary antiphospholipid syndrome associated with anti-phospholipase A2 receptor antibody-positive membranous nephropathy

**DOI:** 10.1186/s12882-020-01856-z

**Published:** 2020-05-24

**Authors:** Maxime Teisseyre, Hélène Perrochia, Pascal Reboul, Sylvain Cariou, Sophie Renaud, Cédric Aglae, Olivier Moranne

**Affiliations:** 1 Nephrology, dialysis and apheresis unit, University of Montpellier-Nimes, CHU de Nîmes, Rue du Professeur Robert Debré, Nîmes, France; 2 Laboratory of anatomopathology, University of Montpellier-Nimes, CHU de Montpellier, Montpellier, France

**Keywords:** Glomerulonephritis, Nephrotic syndrome, Membranous nephropathy, PLA2R antibody, Antiphospholipid syndrome

## Abstract

**Background:**

The kidney is a major target in primary antiphospholipid syndrome. Several types of nephropathy have been reported, the most frequent being acute or chronic specific vascular nephropathies and membranous nephropathy.

**Case presentation:**

A 59-year-old male presented in our unit with nephrotic syndrome. He had a history of primary antiphospholipid syndrome with lupus anticoagulant treated with vitamin K antagonist therapy. On admission, antiphospholipid (lupus anticoagulant) and anti-PLA2R antibodies were positive. Screening for secondary etiologies was negative. In the context of primary antiphospholipid syndrome treated with vitamin K antagonist therapy, we did not perform a biopsy and we treated the patient with angiotensin-converting-enzyme inhibitor. No remission was observed at 6 months with persistent anti-PLA2R antibodies while antiphospholipid antibody level became negative. Consequently, kidney biopsy was performed showing both membranous nephropathy with PLA2R in deposits on immunohistochemistry with IgG4 dominance and antiphospholipid syndrome chronic vascular nephropathy. Following that, treatment with rituximab was started with secondarily a decrease in serum PLA2R antibody levels and partial remission.

**Conclusion:**

We report the first association between primary antiphospholipid syndrome and membranous nephropathy with anti-PLA2R antibodies. Our observations could suggest a causal link between primary antiphospholipid syndrome and PLA2R-related membranous nephropathy. Consequently, it would be interesting to screen for anti-PLA2R antibodies for further cases of nephrotic syndrome in patients with primary antiphospholipid syndrome and to search antiphospholipid antibodies in all membranous nephropathies.

## Background

Antiphospholipid syndrome (APS) is a systemic autoimmune disorder defined by the association of venous and/or arterial thrombosis and/or pregnancy complications and the presence of antiphospholipid antibodies. APS can be isolated (primary antiphospholipid syndrome) or associated with other autoimmune diseases, such as systemic lupus erythematosus (SLE).

The kidney is a major target in primary APS with several types of nephropathy reported. The most frequently reported are vascular nephropathies. The vascular nephropathy in primary APS is classified as either acute or chronic depending on light microscopy findings. Acute vascular nephropathy is diagnosed by a thrombotic microangiopathy, whereas the chronic version presents arteriosclerosis (75%), fibrous intimal hyperplasia (75%), tubular thyroidization (75%), arteriolar occlusions (68%) and focal cortical atrophy (62%) [1]. In a large series of patients with APS [2], thrombotic renal complications occurred only in 2.7% of cases. Another study by Fakhouri et al. [3] identified 29 biopsies performed in patients with APS. In 9 cases, predominant pathological features distinct from vascular APS nephropathy were noted, especially membranous nephropathy (MN) (3 cases).

MN is a non-inflammatory autoimmune disease which affects the glomerulus. It can be primitive or secondary to various pathologies such as autoimmune disease.

Anti-PLA2R antibodies are highly specific to primary MN, however anti-PLA2R antibodies have been identified in some secondary MN such as hepatitis B (HBV) and C (HCV), use of non-steroidal anti-inflammatory drugs, solid tumor and sarcoidosis [4].

## Case report

We report a case of a 59-year-old male hospitalized in May 2017 for nephrotic syndrome, discovered after systematic dipstick. The patient’s past medical history was primary APS with lupus anticoagulant confirmed at 12-week interval, discovered in June 2015 after a second pulmonary embolism. No criteria for SLE were present. His usual treatment was vitamin K antagonist therapy.

On admission, he presented edema of the lower limbs; normal blood pressure; creatininemia 1.7 mg/dl (150 μmol/l); albuminemia 25.9 g/l; albuminuria 4.7 g/g; microscopic hematuria; normal renal ultrasound; negative anti-nuclear antibodies; positive lupus anticoagulant; and anti-PLA2R titer of 1:320 on indirect immunofluorescence, confirmed later on ELISA. HIV, syphilis, HBV and HCV serologies and screening for malignancy were negative.

Due to the high recurrence risk of pulmonary embolism when stopping anticoagulation, we did not perform a biopsy and we treated the patient as an idiopathic MN with angiotensin-converting-enzyme inhibitor. Three months after the onset of nephrotic syndrome, antiphospholipid antibodies spontaneously became negative. No remission was observed at 6 months with angiotensin-converting-enzyme inhibitor treatment with albuminemia 38 g/l, albuminuria 6.4 g/g and persistent anti-PLA2R antibodies.

**Fig. 1:**
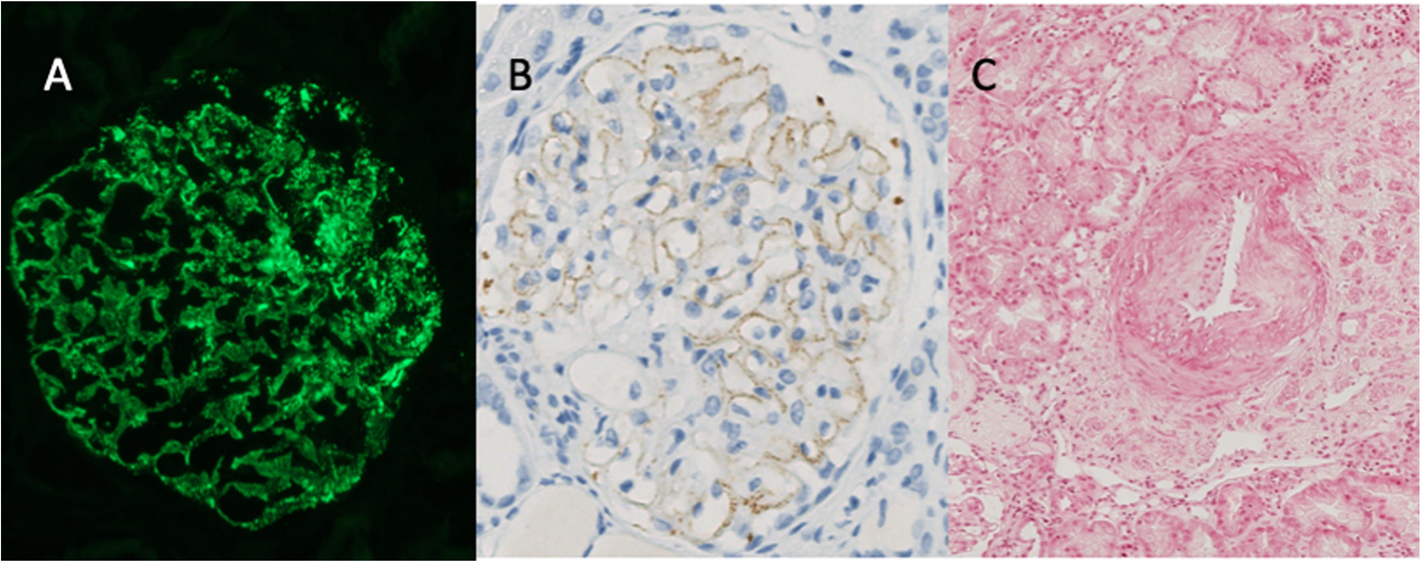
patient’s kidney biopsy. **a** Staining for IgG4 on immunofluorescence (× 400). **b** PLA2R-antigen on immunochemistry (× 400). **c** Primary antiphospholipid syndrome chronic vascular nephropathy with asymmetric fibrous intimal hyperplasia (Periodic-Acid-Schiff X400)

Consequently, kidney biopsy was performed showing both i) antiphospholipid syndrome chronic vascular nephropathy with asymmetric fibrous intimal hyperplasia and tubular thyroidization and ii) MN stage II with diffuse glomerular basement membrane thickening on light microscopy and “spikes” on silver stain, without cellular proliferation. Immunofluorescence microscopy revealed a diffuse granular pattern of C3 and immunoglobulin G (with IgG4 dominance) staining along the glomerular basement membrane (staining for IgA, IgM and C1q were negative). Immunohistochemistry revealed PLA2R in deposits (Fig. 1).

A treatment with rituximab was started 6 months after the beginning of follow-up (2 infusions of 375 mg/m^2^ administered 1 week apart) with 9 months later a decrease in serum PLA2R antibody levels at a titer of 1:10 on indirect immunofluorescence and partial remission.

## Discussion

To our knowledge, we report the first case of a patient who was diagnosed with primary APS associated with APS chronic vascular nephropathy and MN with anti-PLA2R antibodies without any features of lupus MN.

The etiology of this association is unclear. One hypothesis is that antibodies appear against a dysimmunitary background and generate a multisystem autoimmune syndrome. For example, Stehlé et al. [4] showed, in 9 patients who had MN and sarcoidosis, that all patients with active sarcoidosis had PLA2R antigen in deposits, while none of those with inactive sarcoidosis had detectable PLA2R in biopsy. The diagnosis of sarcoidosis could precede the diagnosis of MN by several years. In 2 cases, sarcoidosis activity was linked to the evolution of serum PLA2R antibody levels and proteinuria. Therefore, they hypothesized that there was a causal link between the two diseases and that the immunological setting of sarcoidosis might trigger immunization against PLA2R. Thus we might also ask if the immunological setting of APS might trigger immunization against PLA2R. In our case the APS was quiescent under treatment in recent years but the antiphospholipid antibodies (lupus anticoagulant) were positive at the onset of nephrotic syndrome. Three months after the onset of nephrotic syndrome (prior to treatment with rituximab) these antibodies spontaneously became negative, and partial remission as well as a decrease in serum PLA2R antibody levels occurred 12 months later. Partial remission of the nephrotic syndrome and decrease in serum PLA2R antibody levels could therefore be related to the lack of APS immunological activity.

Another hypothesis is that MN is the first event of SLE, which is one of the main etiologies of secondary APS. Indeed, Sinico and co-workers [5] studied the prevalence and clinicopathologic features of renal involvement in 160 patients with primary APS. Renal involvement was observed in 14/160 (9%), of whom 4/14 patients (28.5%) suffered from MN with atypical features usually related to lupus MN (mesangial proliferation, mesangial and/or subendothelilal deposits, C1q deposits and IgA deposits). Regrettably, PLA2R antibody assay was not reported. Despite a long follow-up (6.9 up to 14.1 years), none developed full-blown lupus. It should be noted that these patients were treated with steroids (plus cyclophosphamide in 2 patients) because of renal disease, and this could have prevented other lupus manifestations from occurring. As MN with atypical features may precede the clinical and biologic manifestations of SLE, these patients could eventually develop SLE and need long-term follow-up. However, in our observation, the presence of anti-PLA2R antibodies and PLA2R in deposits makes the diagnosis of lupus MN unlikely and no sign of SLE was observed after 4 years of follow-up.

Finally, a fortuitous association cannot be eliminated.

In our patient’s case, the presence of anti-PLA2R antibodies motivated the use of rituximab, which may explain the partial remission of the nephrotic syndrome. Our patient’s prognosis is probably worse than PLA2R-related MN without primary APS due to the presence of antiphospholipid syndrome chronic vascular nephropathy with advanced chronic vascular lesions.

To conclude, we observed the first association between primary APS and PLA2R-related MN. Our observations could suggest a causal link between primary antiphospholipid syndrome and PLA2R-related MN. Consequently, it would be interesting to screen for anti-PLA2R in further cases of nephrotic syndrome in patients with APS and to search APS antibodies in all MN.

## Data Availability

The datasets used and/or analyzed during the current study are available from the corresponding author on reasonable request.

## Abbreviations

APS: Antiphospholipid syndrome
HBV: Hepatitis B virus
HCV: Hepatitis C virus
HIV: Human immunodeficiency virus
IgG: Immunoglobulin G
MN: Membranous nephropathy
PLA2R: Phospholipase A2 receptor
SLE: Systemic lupus erythematosus
